# Minimally Invasive Surgical Techniques for Management of Painful Metastatic and Primary Spinal Tumors

**DOI:** 10.7759/cureus.1114

**Published:** 2017-03-24

**Authors:** Omid Hariri, Ariel Takayanagi, Dan E Miulli, Javed Siddiqi, Frank Vrionis

**Affiliations:** 1 Department of Neurosurgery, Riverside University Health System Medical Center, Moreno Valley, California, United States; 2 Department of Neurological Surgery, Marcus Neuroscience Institute, Boca Raton, Florida, United States

**Keywords:** kyphoplasty, vertebroplasty, myeloma, percutaneous, spine, tumors, palliative, metastases, minimally invasive, augmentation

## Abstract

Patients with metastatic spinal disease are affected by disabling pain. The treatment of spinal metastases is focused on pain reduction and improvement in quality of life. Until recently, many patients with metastatic spinal disease did not qualify as surgical candidates due to the risks of surgery and length of recovery period. However, recent advances in minimally invasive surgery such as kyphoplasty and vertebroplasty allow patients to safely undergo surgery for pain relief with a short recovery period.

The studies reviewed here suggest that vertebral augmentation is successful in reducing pain and disability scores in patients with painful metastases and multiple myeloma and are a safe modality to provide lasting pain relief. As the use of kyphoplasty and vertebroplasty for treatment of vertebral metastases is becoming more common, new combinations of cement augmentation with other techniques such as percutaneous pedicle screws and radiofrequency ablation are being explored. The implementation of kyphoplasty and vertebroplasty, in conjunction with other minimally invasive surgical techniques as well as nonsurgical modalities, may lead to the best palliative management of cancer patients with spinal metastases and help them ultimately achieve a better quality of life.

## Introduction and background

### Introduction

Pain from the invasion of cancer into the spinal column is a key detriment to the quality of life of cancer patients. Treatment is focused on pain reduction and quality of life rather than curing the disease process. Multimodal and multidisciplinary treatment is necessary in these patients and includes radiation, medical management, and a variety of operative measures.

While medical treatment may be effective in managing pain for some patients, mechanical pain due to pathological fractures may require surgical intervention. Traditionally, neurological symptoms have been treated by decompression of the spinal cord with a laminectomy. However, many patients with spinal tumors are at a higher risk of complications with open surgery [1]. Recent advances in percutaneous procedures such as kyphoplasty and vertebroplasty have created options for patients who were previously not considered surgical candidates.

Here we provide a brief review of the pathophysiology and clinical symptoms of vertebral tumors along with diagnostic and nonsurgical treatment strategies. We then review percutaneous surgical treatment with an emphasis on the efficacy and safety of vertebroplasty and kyphoplasty.

### Background

The metastatic neoplasms that most commonly present with spinal cord compression are breast (in 22% of patients with breast cancer), lung (15% of patients with lung cancer), and prostate (10% of patients with prostate cancer) [2]. Secondary tumors of the spine most frequently occur in the thoracic spine (70%), followed by the lumbar spine (20%), and finally the cervical spine (10%) [3].

Several theories exist regarding the pathogenesis of vertebral metastases. The 1940 Batson study on cadavers suggested cancer cells are pushed into a valveless venous plexus from the chest and pelvis during periods of increased intra-abdominal pressure. The low-pressure venous system and repeated reversal of flow likely allow for cancer cells to be lodged in the vertebral bodies [4]. A more recent study by Arguello, et al. suggests the tumors alternatively metastasize through arterial circulation rather than through venous routes and use the bone marrow as a “soil for proliferation” [5]. Tumors can also invade locally into the vertebral bodies [6] from retroperitoneal lymph node, lung (Pancoast tumors), thyroid, or muscle (sarcomas) metastases.

While metastases are the most common form of cancer in the spine, multiple myeloma is the most common primary tumor to invade the vertebrae. While multiple myeloma is a “primary” neoplasm in the sense that it begins in the bone marrow, it is derived from plasma cells rather than osteocytes like many of the other bony primary cancers.

Multiple myeloma patients are especially at risk for pathological fractures due to the combination of local invasion of malignant myeloid cells and the cytokine-mediated activation of osteoclasts that causes an increase in bone resorption [7]. In these patients, 80% of pathologic vertebral fractures occur from T6-L4, and 50% from T11-L1 [8]. Involvement of the cervical spine is uncommon [9].

## Review

### Clinical presentation

The most common symptom at the time of diagnosis is severe pain, which usually precedes the onset of neurologic dysfunction by a median time of seven weeks [10-12]. The main types of pain experienced in these patients are local pain and axial pain.

Local pain occurs at the site of the metastasis, is severe and progressive in quality [13], and is described by patients as “gnawing” or “aching” [14]. This pain classically presents nocturnally secondary to its exacerbation by supine positioning, is relieved by standing up, and can often be elicited by percussion over the affected region [10]. Local pain is caused by inflammation and stretching of the periosteum where nociceptors are located [14]. Because back pain caused by other conditions (e.g., degenerative joint disease) typically occurs in the cervical and lumbar regions, patients with thoracic pain should be treated with a high index of suspicion for spinal metastases [10].

Mechanical or axial pain typically appears later than local pain and can occur secondary to vertebral instability and compression of the spinal cord. This pain is described as sharp and stabbing [10], is exacerbated by axial loading [15], and usually persists despite pain medication [6]. Regardless of the location, mechanical back pain reported by a patient diagnosed with cancer should be assumed to be due to spinal metastases until proven otherwise [1]. Axial pain can be treated at an early stage with surgical techniques such as vertebroplasty and kyphoplasty as described later.

After pain, the second most common symptom of metastatic spinal disease is neurologic dysfunction secondary to spinal cord compression. Sixty percent to 85% of patients have corticospinal dysfunction, presenting with upper motor neuron disease. Loss of sensation occurs along with (or soon after) the onset of weakness [11]. Patients commonly present with difficulty in walking due to hip flexor weakness, and soon afterward complain of changes in sensation. Autonomic dysfunction can also occur as a late symptom, usually presenting as urinary incontinence [11].

Similarly, multiple myeloma patients experience pain secondary to invasion of the periosteum which causes local pain and weakening of the vertebral body. The multiplicity of pain sites due to the diffuse involvement of numerous vertebrae is the rule with multiple myeloma.

### Radiographic findings

X-ray is typically the first imaging modality used for patients with vertebral metastases. Plain anteroposterior and lateral radiographs may reveal asymmetry with areas of radiolucency or opacity, depending on the type of lesion present. The classic “winking owl sign” can be seen on anteroposterior plain films due to a missing pedicle, but requires significant bone destruction to be visible. When an abnormality is present on an x-ray, computed tomography (CT) is recommended to visualize the bone abnormalities at higher resolutions [15].

While x-ray and CT are important in assessing bone involvement, magnetic resonance imaging (MRI) with gadolinium enhancement is the gold standard imaging modality for suspected spinal metastases. MRI has a high sensitivity for tumors using sagittal T1 or short T1 inversion recovery. T2 sagittal and axial T1 or T2 are useful in detecting soft tissue involvement and determining the degree of spinal cord compression [12]. Moreover, focal vertebral lesions in multiple myeloma present with hypointense on T1 and hyperintense on T2 [8].

### Nonsurgical pain management

There are several nonsurgical methods for managing spinal pain in cancer patients. The 1997 World Health Organization (WHO) pain ladder remains the standard for the medical management of pain. Patients are categorized as having mild to moderate or moderate to severe pain and are treated with the corresponding medications. Mild pain is managed with nonopioid analgesics such as nonsteroidal anti-inflammatory drugs (NSAIDs) and acetaminophen, while moderate pain is managed with weak opioids such as dihydrocodone or tramadol. NSAIDs may be added at any level on the analgesic ladder. Oral morphine is the drug of choice for treating the severe pain experienced by patients with metastatic spinal lesions [16-17]. Patients should receive continuous dosing with the administration of extra doses for breakthrough pain [16].

Treatment of pain following the WHO guidelines is successful in reducing pain by 70% to 80% [18]. Local nerve blocks, antidepressants, anticonvulsants [19], and intrathecal analgesia [12] can be used as adjuvant treatment depending on whether the pain is local, radicular, or axial.

Corticosteroids can also reduce pain in patients with spinal metastases by inhibiting prostaglandin synthesis and decreasing vascular permeability. Pain is abated by reducing edema in the surrounding tissue, which prevents the compression of pain-producing structures [12]. Dexamethasone is commonly used because of its longer half-life, once-a-day-dosing, and higher potency [18].

Radiotherapy can also be used to decrease pain from spinal metastases, especially in conjunction with surgery. Radiotherapy has become the standard of treatment of patients with multiple metastatic lesions who are not surgical candidates due to poor prognosis and severe comorbidities. However, recent advances in percutaneous surgery indicate the benefits of these procedures may outweigh the risks [19].

In a prospective study of metastatic cancer pain treated with radiation therapy, 49% of patients experienced complete pain relief while 51% had a >50% reduction in pain [20]. While high-dose radiation can provide pain relief, it increases the risk of vertebral compression fractures due to osteonecrosis [19]. Fracture risk must be weighed against the efficacy of pain relief when determining stereotactic radiation treatment regimens. In selected cases, biopsy, kyphoplasty, and radiofrequency ablation can be performed prior to radiosurgery. In addition to dosage, the frequency of radiation also has an effect on treatment outcomes. Although both split-course and short-course regimens have similar efficacy, short-term therapy is more practical and requires fewer patient visits [21].

Management of axial pain is similar for multiple myeloma in that the WHO guidelines are used to manage pain medically. The most commonly used treatment of multiple myeloma is chemotherapy, while the definitive treatment requires autologous hematopoietic stem cell transplant [22].

### Surgical techniques

Medical treatments may help significantly with local pain; however, they do not address mechanical spinal pain caused by vertebral collapse and deformity [6]. Surgical treatments, on the other hand, have been shown to be highly effective in treating axial pain [23] with few adverse effects. Vertebroplasty and kyphoplasty offer minimally invasive surgical options for patients who are unlikely to tolerate more extensive invasive procedures [19].

**Figure 1:**
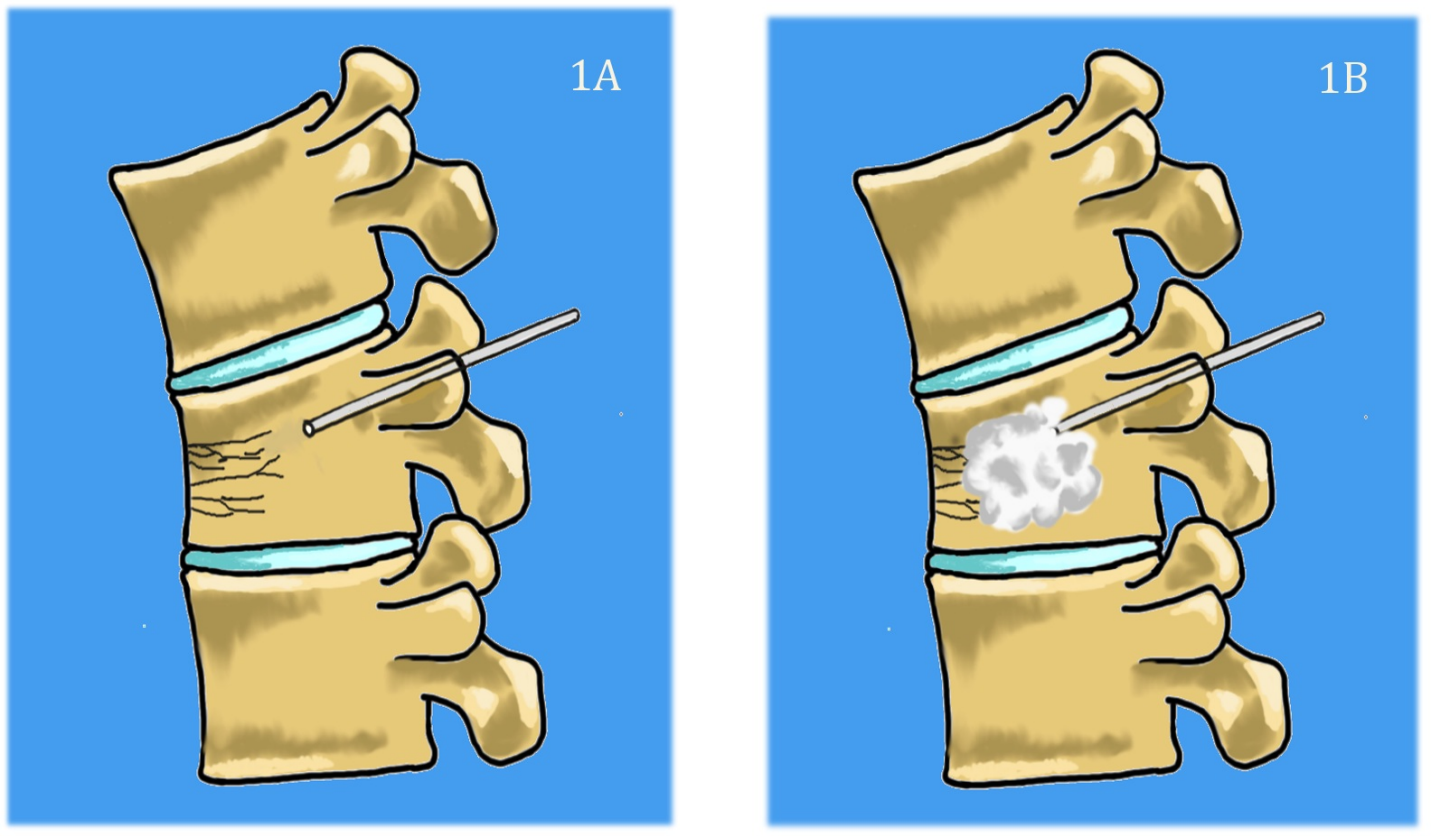
Basic steps of vertebroplasty technique. 1A) Needle is inserted percutaneously through the pedicles and into the vertebral body. 1B) polymethylmethacrylate (PMMA) cement is then injected into the vertebral body to relieve mechanical stress and restore height.

**Figure 2:**
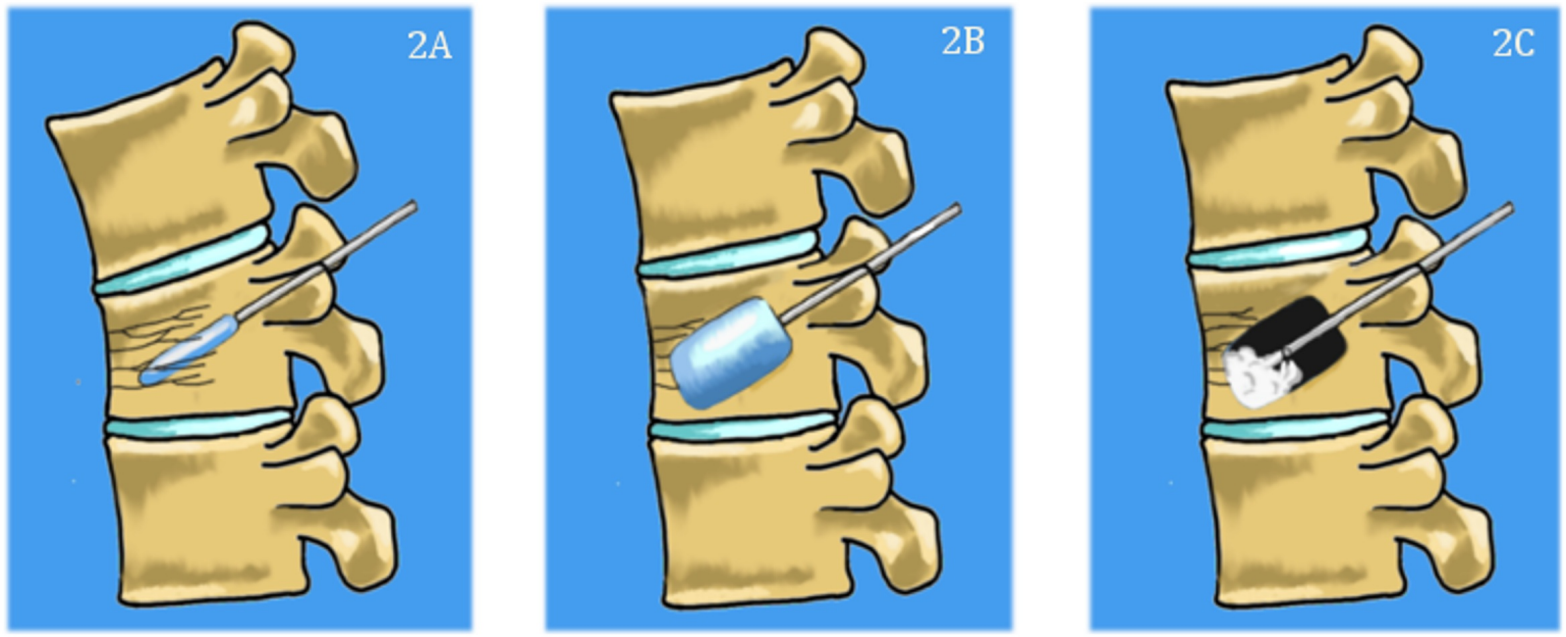
Basic steps of kyphoplasty. The needle is inserted percutaneously, and then a balloon is inserted (2A) and inflated (2B) to create a cavity inside the vertebral body and to restore height. Cement is then injected into the cavity (2C).

Kyphoplasty and vertebroplasty are both effective in relieving axial pain, but no consensus has been made on when to implement one over the other [24]. While the type of procedure is often determined by the surgeon and institutional preference, it may be valuable to develop a more standardized treatment algorithm in the future. Figures 1-2 describe the two procedures.

**Figure 3:**
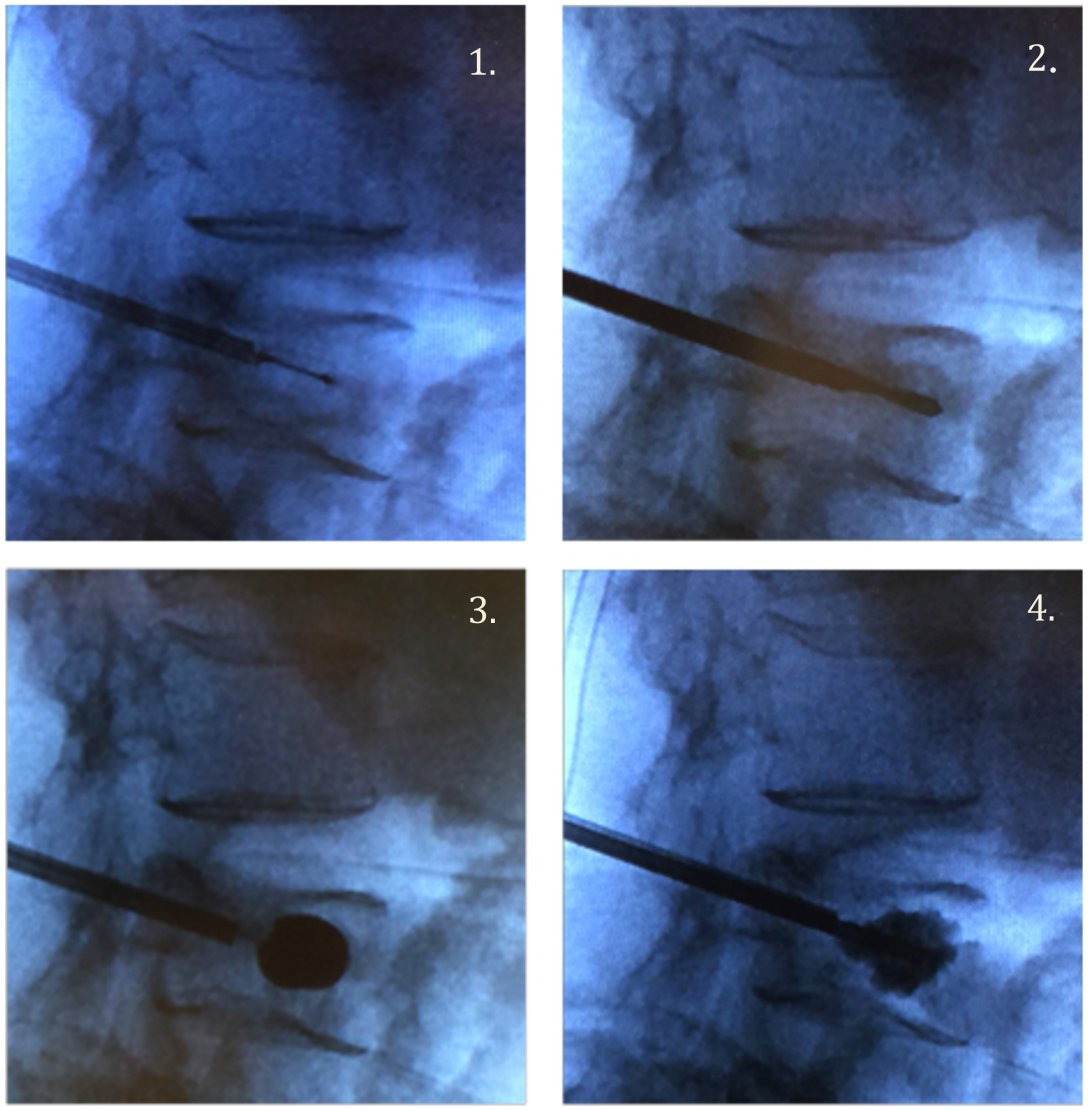
Intraoperative films during kyphoplasty of L4. 1. Insertion of needle into anterior one-third of vertebral body. 2. Replacement of the needle with the balloon. 3. Inflation of the balloon. 4. Injection of polymethylmethacrylate (PMMA) cement.

Vertebroplasty and kyphoplasty have traditionally been performed in the lumbar and lower thoracic spine, using a transpedicular approach as shown in Figure 2. Intraoperative x-rays of a lumbar kyphoplasty are shown in Figure 3. Although kyphoplasty and vertebroplasty may be more difficult in the cervical spine due to the anatomy of the cervical vertebrae and the proximity of the cervical vasculature, these techniques have been successfully applied to the cervical spine. A meta-analysis of six studies included 120 patients who underwent vertebroplasty or kyphoplasty for metastases to the cervical spine and showed significant reductions in mean pain scores from 7.6 ± 0.9 preoperatively to 1.9 ±.8 (p = .0006) at the final assessment (range: three months to 21.8 months after surgery) [25]. Twenty-two (16%) of 120 patients experienced asymptomatic cement leaks, while there were three cases of odynophagia, one case of stroke, and one case of occipital neuralgia secondary to cement leakage [25].

The upper thoracic spine also proves to be difficult for kyphoplasty due to the small pedicles. An extra-pedicular approach has been developed for upper thoracic spinal metastases, which involves inserting the needle into a plane between the proximal rib and transverse process at the lateral wall of the pedicle, and then traversing the needle through the transverse process, costotransverse joint, and rib to access the vertebral body. This requires extensive expertise and skill to implement successfully and may have a higher risk of complications. A recent study of kyphoplasty using an extra-pedicular approach in 14 patients with metastatic disease of the upper thoracic spine showed significant improvements in mean visual analogue scale (VAS) scores from 79 preoperatively to 30 postoperatively (p < .001), and mean Oswestry disability index (ODI) scores from 83 preoperatively to 33 postoperatively [26].

### Efficacy of kyphoplasty and vertebroplasty for metastatic neoplasms

Fourney, et al. [24] studied the efficacy and safety of vertebroplasty and kyphoplasty in 56 patients with either spinal metastases or spinal multiple myeloma. Eighty-four percent of these patients reported complete or significant pain relief, with statistically significant improvements in median preoperative and postoperative VAS scores. A summary of the efficacy of kyphoplasty and vertebroplasty in pain reduction and disability scores is demonstrated in Table 1. All significant results in this paper correspond to a 95% confidence interval. Significant pain reduction was maintained at one, three, and six months for patients who underwent kyphoplasty or vertebroplasty. Significant pain reduction was additionally maintained at one year for kyphoplasty but not for vertebroplasty. No significant difference in pain reduction between kyphoplasty and vertebroplasty was noted at six months. There were also no complications related to the procedure in any of the patients in the trial [24]. This was the first large study to assess the safety and benefits of kyphoplasty and vertebroplasty for painful spinal neoplasms. The high percentage of patients with significant pain relief and lack of significant complications suggest that both kyphoplasty and vertebroplasty can provide immediate, safe, and lasting pain relief in these patients.

**Table 1:** Scores for pain, disability, and physical function in patients before and after kyphoplasty and vertebroplasty. †Statistically significant. Abbreviations: Pt, patient; KP, kyphoplasty; VP, vertebroplasty; NRS, pain numeric rating score; ODI, Oswestry disability index; VAS, visual analogue scale; RDQ, Rolland Disability Questionnaire; KPS, Karnofsky performance score; Sig., significant; MM, multiple myeloma; mo, month; NA, not applicable; Postop, postoperatively; Preop, preoperatively; CI, confidence interval.

Study	Median Pt Age	# Pts	Procedure (# cases)	Pathology (#cases)	Pain Relief: improvement in scores postop	Karnofsky Performance Status	Improvement in Disability Scores after KP/VP	Summary
Berenson 2011 [27]: Randomized Controlled Multicenter Trial	KP: 64.8, Control:63	134	KP(68) vs. medical management (61)	Multiple Myeloma: KP (22), nonsurgical (27)	^†^Difference in reduction of NRS score between KP and medical management. KP > medical.	^†^Improvement in KP group compared to nonsurgical group (mean improvement): 15.3 points (95% CI, 13.5 to 17.1; p < .0001)	†RDQ treatment effect after KP: -8.4 points (95% CI, -7.6 to -9.2; p < .0001)	Sig. reductions in mean pain scores (NRS), KPS, RMQ for KP at one month, but not in nonsurgical group.
				Metastatic Cancer: KP (46), medical (34)	1 week: -3.5 (95% CI, -3.8 to -3.2; p < .0001)			
					1 mo.: -3.3 (95% CI, -3.6 to -3.0; p < .0001) l (95% CI, p < .0001).			
Fourney 2003 [24]: Retrospective Review	64	56	KP (34), VP (15), KP+VP (7)	Metastatic cancer (35), Multiple Myeloma (21)	†Improvement in VAS:	NA	NA	Sig. reductions in VAS in KP patients with MM and spinal metastases compared to baseline, with significance maintained at 6 mo. for VP, and at one year for KP.
					immediately postop: BKP, VP (p < .05).			
					6 mo.: KP, VP (p < .05)			
					12 mo.:KP (p < .05)			
Markmiller 2015 [28]: longitudinal prospective case series	68.7	115	Kyphoplasty	Metastatic Cancer (92), Multiple Myeloma (23)	†Median VAS after KP:	†Median:	^†^Improvement in mean ODI after KP:	Sig. improvements in median KPS, mean ODI, and median VAS with KP through 12 mo.
					Postop: -4.0 (95% CI -5.0 to -3.0; p < .0001)	Postop: 15 (95% CI; p < .0001)	Postop: -49.6 (95% CI, -56.4 to -42.1 p < .0001)	
					12 mo.: -3.5 (95% CI -5.0 to -3.0 ( p < .0001)	12 mo: 10 (95% CI; p < .0001)	12 mo.: -48.4 (95% CI, -56.4 to -42.1 p < .0001)	
McDonald 2009 [29]: retrospective review	66.2	67	VP (67)	Multiple Myeloma (67)	^†^median improvement in VAS after VP:	NA	†Improvement in median RDQ scores after VP:	Sig. improvements in median VAS, RDQ in patients with MM after VP through 12 mo.
					At rest: -2.7 (95% CI, -3.7 to 1.7; p < .0001 at one week, < .03 at one year)		one week: -11 (95% CI -14.3 to -7.7; p < .0001)	
					With activity: -5.3 (95% CI, -6.4 to -4.2 p < .0001 at one week, p < .001 at one year)		one year: (p < .001)	
Papanastassiou 2014 [31]: retrospective comparative study	61.6	69	KP: unilateral versus bilateral (69)	Multiple Myeloma (69)	^†^Change in mean VAS in unilateral and bilateral KP, respectively from baseline	NA	NA	Sig. improvement in mean VAS scores in patients with MM after both unilateral and bilateral kyphoplasty, with no difference in pain reduction between the two techniques.
					Postop: -5.4, -5.5(95% CI, p<.0005)			
					3 mo.: p<.0005			
Pflugmacher 2006 [30]: Longitudinal prospective case series	62.4	20	KP(20)	Multiple Myeloma (20)	†Change in mean VAS:	NA	†Improvement in mean ODI:	Sig. improvements in mean VAS, ODI with KP in patients with multiple myeloma through one year.
							Preop: 71.5% (39-89%)	
					Postop: -6 (95% CI; p < .05)		3 mo. postop: 27.5% improvement 27.5% (95% CI, 11 to 41%; p < .05)	
					12 mo.: -5.1 (95% CI; p < .05)		12 mo.:(95% CI, 13 to 52%; p < .05)	

The Cancer Patient Fracture Evaluation (CAFÉ) trial by Berenson, et al. [27] was the first randomized controlled multicenter study that compared the treatment outcomes of kyphoplasty and nonsurgical treatment in patients with painful vertebral fractures due to metastases. One hundred thirty-four patients were enrolled.

Assessment after one month showed statistically significant improvements in physical function, back pain, and quality of life in the kyphoplasty group; the effects were not statistically significant for the nonsurgical treatment group. The mean Roland Morris disability questionnaire (RDQ) treatment effect for kyphoplasty was statistically significant (Table 1). Mean Karnofsky performance scores (KPS) also increased significantly, showing an improvement in functional impairment status that was not seen in the nonsurgical treatment group. Kyphoplasty patients experienced an improvement in quality of life both physically and mentally, as demonstrated by significant improvements in mean Short Form Health Survey (SF-36) Physical Component Summary scores and SF-36 Mental Component Summary scores at one month. Reduction in back pain was assessed using the numeric pain rating scale (NRS), with a significant difference in reduction between kyphoplasty and nonsurgical groups at one week and one month. Thirty-eight patients in the nonsurgical group elected to receive kyphoplasty when given the option after one month [27].

This trial was the first randomized controlled trial to assess the efficacy of kyphoplasty in patients with painful spinal metastases. Although randomization was only sustained for follow-up at one month, results suggest that kyphoplasty is an effective palliative care strategy for patients with painful spinal metastases, improving quality of life, functional status, and back pain.

Markmiller [28] recently published a longitudinal prospective case series of 115 patients who received kyphoplasty for confirmed spinal metastases or multiple myeloma. At discharge after kyphoplasty, a statistically significant decrease in median VAS scores was seen compared to preoperative scores; this remained significant at one year (Table 1). Similarly, median KPS scores improved significantly from preoperative scores, and improvement in function was still found to be significant at one year. Mean ODI scores showed significant improvements compared to baseline, at discharge, and at one year. These results are consistent with the aforementioned studies, and show potential for lasting pain relief and improved functional status.

### Efficacy of kyphoplasty and vertebroplasty for multiple myeloma

Recently, studies on percutaneous vertebral augmentation for multiple myeloma have emerged. While both metastatic tumors and multiple myeloma invade the vertebral bodies, the pathophysiology of multiple myeloma is drastically different, as previously described. It is important to investigate whether patients with multiple myeloma can benefit from kyphoplasty and vertebroplasty with as much success. McDonald, et al. [29] measured quantitative outcomes of pain, mobility, and function in 67 patients with multiple myeloma who underwent vertebroplasty. Median RDQ scores and VAS scores at rest and with activity improved significantly (Table 1). Both RDQ and VAS scores remained significant at one week, one month, six months, and one year [29]. Similar findings were reported in a smaller study of 20 patients with multiple myeloma who underwent kyphoplasty, with statistically significant reduction of mean VAS and ODI scores. Improvement in both VAS and ODI scores remained significant at the one-year follow-up [30]. These results suggest that vertebroplasty can improve pain, mobility, and function in multiple myeloma patients as it does in patients with metastatic spine disease.

In a study comparing unilateral versus bilateral kyphoplasty in multiple myeloma patients, patients who received either form of kyphoplasty experienced improvement in pain scores. VAS scores improved from baseline by 30% (p < .0005, paired samples t-test). There was, however, no significant difference in reduction of mean VAS scores between unilateral and bilateral vertebroplasty. The results of this uniform cancer population refute previous notions that bilateral kyphoplasty is more effective in pain resolution than a unilateral approach. The authors recommend the use of unilateral kyphoplasty whenever feasible to reduce operation time and risks of surgery, especially because patients with multiple myeloma often require cement augmentation on multiple levels [31].

There is no consensus in the literature on the maximum number of adjacent levels that can be treated in one session. A study compared the biomechanics of multilevel polymethylmethacrylate (PMMA) vertebral augmentation in cadavers [32]. Zero, one, two, and three levels were augmented and stiffness and strength were analyzed using a univariate analysis of variance. Stiffness (p = .0009 multilevel segments, p < .0004) and strength (p < .001) depended on bone marrow density but did not significantly differ among the groups with zero, one, two, or three levels treated. This study suggests that the strength of vertebral bodies is not negatively affected by an increased number of adjacent levels treated with kyphoplasty or vertebroplasty. Although some sources suggest that no more than two levels should be augmented due to possible PMMA toxicity, many experts in the field [33] have recommended treating up to three levels.

### Safety of vertebroplasty and kyphoplasty in metastatic spinal disease

The main concern with kyphoplasty and vertebroplasty is the potential for PMMA cement leakage. Possible but seldom seen complications secondary to cement leakage include spinal cord compression, radiculopathy, and pulmonary embolism. One-third of patients in Markmiller, et al. experienced cement leakage; however, only three (2.6%) were symptomatic with resolution after three months [28]. In Fourney, et al., six of 65 patients who underwent kyphoplasty or vertebroplasty for metastases showed leakage of cement on imaging, but none were symptomatic [24]. Leakage rates are compared in Tables 2-3.

**Table 2:** Cement leakage in studies reporting number of levels with leakage. Abbreviations: Pts, patients; KP, kyphoplasty; VP, vertebroplasty.

Study	Patients	Procedure	Pathology (#pts)	# Levels with leakage (% total # levels treated)	Location of Leakage: # of levels (% total number of levels treated)	Total # patients with symptomatic leakage (% total number pts)
Fourney 2003 [24]	56 pts	KP (34 pts)	Metastatic cancer (35), Multiple Myeloma (21)	6 levels (9.2%)	disc: 5 (6.7% levels after VP)	0
	97 levels:	VP (15 pts)				
	65 VP	KP+VP (seven patients)			anterior paravertebral soft tissue: 1 (1.5% levels after VP)	
	32 KP					
Pflugmacher 2006 [30]	20 pts	KP	Multiple Myeloma (20)	5 levels (10.4%)	disc: 3 (6.25%)	0
	48 levels				paravertebral: 2 (4.1%)	

**Table 3:** Cement leakage in studies reporting number of patients with leakage. *Three patients with symptomatic leakage. All three patients experienced radiculopathy with no weakness and had complete resolution of symptoms at six months. Two patients with leakage into medullary canal, one patient with paravertebral leakage. Abbreviations: KP, kyphoplasty.

Study	Patients	Procedure	Pathology (# cases)	# Patients with leakage	Location of Leakage: # patients (% patients)	Total patients with symptomatic leakage (% total number patients)
Markmiller 2015 [28]	115 patients	KP	Metastatic Cancer (92), Multiple Myeloma (23)	40 patients (34.8% of 115 patients)	disc: 17 (14.8%)	3 (2.6%)*
					disc-paravertebral: 2 (1.7%)	
					medullary canal: 8 (7%)	
					paravertebral: 9 (7.8%)	
					vascular: 4 (3.5%)	
McDonald 2009 [29]	67 patients	KP	Multiple Myeloma (67)	13 patients (19% of patients)	disc: 6 (9%)	0
					paravertebral 4 (6%)	
					embolus to epidural vein: 3 (4%)	
Papanastassiou 2014 [31]	69 patients, 105 levels	KP	Multiple Myeloma (69)	five patients (7% patients)	disc, spinal canal	0

Although symptomatic cement extrusion is uncommon, there are several strategies to prevent leakage. Fourney, et al. suggest that their low leak rate may be due to dedicated time for sufficient thickening of the cement before injecting into the vertebral body. Another strategy is to use smaller amounts of PMMA cement [24].

### Safety of vertebroplasty and kyphoplasty in patients with multiple myeloma

In McDonald, et al. [29], 13 of 67 patients (19%) who underwent vertebroplasty experienced the injection of cement into areas outside of the vertebral body, but all were asymptomatic [29]. The lack of symptomatic complications from the procedure suggests vertebroplasty is a safe treatment modality for multiple myeloma patients in addition to patients with spinal metastases (Tables 2-3). Similar recommendations were made in a study of kyphoplasty in patients with spinal metastases and multiple myeloma. Because patients with multiple myeloma of the spine experienced increased cement leakage rates due to softer integrity of the bone, the authors suggested waiting for cement to become more viscous to prevent leakage [30].

### Efficacy of minimally invasive options: percutaneous pedicle screws combined with vertebral augmentation

In patients with involvement of the pedicle and posterior elements, cement augmentation may not be adequate treatment. Moreover, open resection, decompression, and fusion can be morbid secondary to steep recovery in late-stage cancer patients. In patients who require additional stabilization but cannot tolerate open surgery, percutaneous pedicle screw fixation may be considered. In a study by Chi, et al. in 2013 [34], 16 patients with pathologic fractures secondary to spinal metastases underwent pedicle screw fixation using fluoroscopic guidance. In 14 of 16 patients, vertebroplasty was performed following screw fixation. Pain significantly decreased postoperatively as measured by the numeric pain rating scale (p < .01). Patients also had a significant improvement in kyphotic angle (p < .01). Similar to kyphoplasty or vertebroplasty alone, the percutaneous treatment of instability allows patients to recover quickly and start or return to radiotherapy or chemotherapy shortly after surgery. The combination of percutaneous pedicle screw fixation and vertebral augmentation is perhaps an option in patients with significant neoplastic destabilization who will not tolerate more extensive and open surgical approaches.

Zairi, et al. [35] studied 10 patients with spinal metastases who experienced neurological compromise and underwent minimally invasive transpedicular vertebrectomy with spinal cord decompression and subsequent percutaneous stabilization. Eight of 10 patients improved at least one Frankel grade, and all patients experienced a reduction in pain using the VAS score. Patients received radiotherapy or chemotherapy. This approach allows for tumor control because radiation and chemotherapy can be initiated almost immediately after surgery without concern of wound dehiscence.

One concern of pedicle fixation is the risk of screw pullout due to the poor bone quality and lack of strong purchase of the pedicle screws in the bone. Moussazadeh, et al. [36] combined two procedures to address this issue. Forty-four patients with spinal instability due to vertebral metastatic tumors underwent percutaneous short-segment pedicle screw fixation with cement augmentation at the affected levels. Transpedicular cement augmentation was followed by screw placement into the cement. Subsequently, kyphoplasty was done at the level of the fracture, and rods were secured to the pedicle screws. Twenty-nine of 44 patients reported complete resolution of symptoms, 13 reported mild pain, and two reported moderate pain after surgical intervention, with a significant decrease of pain on the Serlin scale (p < .001). There were few complications, with one adjacent-level fracture, and one asymptomatic screw pullout. The cement augmentation in the levels with screw fixation allowed the pedicle screws to be fixed more securely, while kyphoplasty at the level of the fracture created better anterior stabilization and prevented future kyphotic deformity [36]. The combination of percutaneous pedicle screw fixation and vertebral augmentation is perhaps an option in patients with significant neoplastic destabilization who will not tolerate more extensive and open surgical approaches.

Gu, et al. [37] demonstrated the benefits of minimally invasive pedicle screw fixation and percutaneous vertebroplasty followed by neurologic decompression and partial tumor resection using a mini-posterior-midline approach. In the study, 18 patients with spinal cord compression due to vertebral tumors were treated. One year after the operation, the median VAS scores decreased significantly from nine preoperatively to three (p < .001). Four patients presented with complete loss of motor function (ASIA scale B), and 14 presented with incomplete motor paralysis (ASIA scale C or D). All patients experienced improvement in paraplegia postoperatively, and 13 of 18 improved to ASIA scale E by the one-year follow-up.

### Experimental use of chemotherapeutic agent-eluting acrylic cement in vertebroplasty

Experimental use of PMMA cement mixed with chemotherapeutic agents is currently being studied. Rosa, et al. [38] showed that methotrexate, cisplatin, and doxorubicin retain biologic effects on in-vitro breast cancer cells when mixed with acrylic cement. However, the amount of drug released decreased to values near zero by 15 days. Maccauro, et al. [39] showed that methotrexate does not weaken the compressive properties of acrylic cement. Possible advantages of adding chemotherapeutic agents to PMMA for vertebroplasty and kyphoplasty include the drug is less likely to be eliminated systemically before reaching its target cells secondary to its local release. Although chemotherapeutic agents seem to be able to retain anti-cancer properties and are unlikely to affect the strength of acrylic cement, the pharmacodynamics of the timing and dosage of the drug must be studied further.

### Radiofrequency ablation and cement augmentation

Recently, the application of radiofrequency ablation (RFA) has been used in tumors that are unresectable and not responsive to radiation therapy. The affected vertebral body is percutaneously accessed, and a high-frequency alternating current is sent to cause thermally-induced necrosis of the tumor. The pathophysiology of pain relief caused by RFA is unclear. One explanation is that it reduces pain by destroying nociceptors as well as reducing the tumor burden [40]. Gevargez, et al. [41] showed that 41 patients treated with RFA for vertebral metastases experienced a significant reduction in VAS scores within six weeks (p = .001) and continued to be significant at six months (p = 0.002).

RFA treatment of tumors within the vertebral body may cause instability if done alone. In a cadaveric study comparing RFA and vertebroplasty with RFA alone, RFA was shown to decrease mechanical stability and increase the risk of subsequent burst fractures [42]. Combining RFA and vertebroplasty provides a way to decrease tumor burden and retain or augment the supportive structure of the vertebral body. Wallace, et al. [40] performed 72 RFA treatments with subsequent vertebroplasty in patients with spinal tumors. Patients treated with this combination of techniques experienced a significant decrease in VAS pain scores at one week and four weeks (median 3.25, 2.75, respectively; p < .0001). Like vertebroplasty or kyphoplasty alone, RFA can result in almost immediate pain relief with a short recovery period [40]. Zheng, et al. [43] showed similar results using RFA combined with kyphoplasty. Thus, RFA may be a safe technique to offer to patients in combination with vertebral augmentation, especially for patients with a large tumor burden. However, further comparative studies are indicated to measure pain reduction in patients treated with percutaneous cement augmentation alone versus RFA.

### Separation surgery and spinal laser interstitial thermal therapy

Separation surgery, as described by Bilsky and Smith [44], is a technique in which a portion of an epidural tumor adjacent to the dura is resected, and instrumentation is done for stabilization without further tumor resection. This technique can be used in patients with radioresistant tumors who would likely benefit from stereotactic radiosurgery. The goal is to decompress the spinal cord and create a safe margin between the thecal sac and the tumor for high-dose radiation therapy while preventing direct damage to the spinal cord. Moreover, it prevents the more aggressive and invasive approaches required when attempting a gross total resection. Therefore, patients proceed to radiation therapy and other adjunct therapy without significant delay [45].

Tatsui, et al. [46] recently described an innovative technique called spinal laser interstitial thermotherapy (SLITT). For select patients, this is an alternative to separation surgery in which the tumor adjacent to the dura is ablated to create a space between the spinal cord and the tumor in preparation for high-dose radiation therapy [46]. A probe is inserted into the epidural tumor percutaneously under CT-based image guidance and advanced to a safe distance from the thecal sac. The tumor is thermally ablated under real-time thermal MRI monitoring to protect the spinal cord from thermal injury [45].

In another study, Tatsui, et al. [47] reported 19 patients with metastatic epidural spinal cord compression who received SLITT experienced a significant decrease in VAS pain scores from 4.72 to 2.56 after one month, which remained significant at three months postoperatively (p = .43 and p = .21, respectively) [47]. All patients were able to undergo subsequent stereotactic radiosurgery (SRS), with a median time from laser ablation to SRS of three days. With one exception, no patients experienced adverse events due to the procedure. The median thickness of the epidural tumor decreased significantly from 8.0 mm preoperatively to 6.4 mm, two months postoperatively (p = .012) [47].

The study demonstrates that SLITT can be performed safely to relieve epidural spinal cord compression with low morbidity in select patients, namely those with radioresistant tumors, and allows for the prompt resumption of adjunct therapy. Like other minimally invasive techniques, this offers a viable alternative for patients who are not candidates for open surgery.

In addition, SLITT can be combined with percutaneous instrumentation in patients who require immediate stabilization [48]. A pilot study of eight patients with epidural spinal cord compression underwent SLITT combined with percutaneous spinal stabilization with a median time to radiation therapy or chemotherapy of five days [48]. Tatsui, et al. showed that thermal MRI guidance for laser ablation has high accuracy and can be used effectively to visualize the spinal cord even when severe spinal cord compression is present [49].

## Conclusions

Patients with painful vertebral metastases and multiple myeloma of the spine must be treated with multiple modalities in a multidisciplinary fashion including but not limited to radiation and medical management. Percutaneous surgical techniques are minimally invasive with high efficacy and a low rate of complications. The studies reviewed here suggest that kyphoplasty and vertebroplasty are successful in reducing pain and disability scores in patients with painful metastases and multiple myeloma. The main concern of these techniques is the extrusion of cement, which has rarely caused lasting neurological symptoms. While kyphoplasty has been implemented traditionally in the lower thoracic and lumbar spine where a trans-pedicular approach can be made, modified techniques to access the vertebral bodies in the upper thoracic spine and cervical spine may allow more patients to benefit from kyphoplasty and vertebroplasty.

Implementation of kyphoplasty and vertebroplasty in conjunction with other minimally invasive techniques as well as nonsurgical modalities may lead to the best palliative management of cancer patients with spinal metastases and help them ultimately achieve a better quality of life.

## References

[1] Chi JH, Gokaslan ZL (2008). Vertebroplasty and kyphoplasty for spinal metastases. Curr Opin Support Palliat Care.

[2] Gerszten PC, Welch WC (2000). Current surgical management of metastatic spinal disease. Oncology.

[3] Klimo P, Schmidt MH (2004). Surgical management of spinal metastases. Oncologist.

[4] Batson OV (1940). The function of the vertebral veins and their role in the spread of metastases. Ann Surg.

[5] Arguello F, Baggs RB, Duerst RE (1990). Pathogenesis of vertebral metastasis and epidural spinal cord compression. Cancer.

[6] Sciubba DM, Gokaslan ZL (2006). Diagnosis and management of metastatic spine disease. Surg Oncol.

[7] La Maida GA, Giarratana LS, Acerbi A (2012). Cement leakage: safety of minimally invasive surgical techniques in the treatment of multiple myeloma vertebral lesions. Eur Spine J.

[8] Tosi P (2013). Diagnosis and treatment of bone disease in multiple myeloma: spotlight on spinal involvement. Scientifica.

[9] Frigui M, Frikha F, Haj Kacem H (2011). Multiple myeloma presenting as cervical spine compression. Rheumatol Rep.

[10] DeAngeles LM, Posner JB (2009). Neurologic Complications of Cancer. https://global.oup.com/academic/product/neurologic-complications-of-cancer-9780195366747?cc=in&lang=en&.

[11] Gabriel K, Schiff D (2004). Metastatic spinal cord compression by solid tumors. Semin Neurol.

[12] (2008). Metastatic Spinal Cord Compression: Diagnosis and Management of Patients at Risk of or with Metastatic Spinal Cord Compression. https://www.ncbi.nlm.nih.gov/books/NBK55007/.

[13] Levack P, Graham J, Collie D (2002). Don’t wait for a sensory level-listen to the symptoms: a prospective audit of the delays in diagnosis of malignant cord compression. Clin Oncol (R Coll Radiol).

[14] Sciubba DM, Petteys RJ, Dekutoski MB (2010). Diagnosis and management of metastatic spine disease. A review. J Neurosurg Spine.

[15] Gebaeur GP, Farfoodi P, Sciubba DM (2008). Magnetic resonance imaging of spine tumors: classification, differential diagnosis, and spectrum of disease. J Bone Joint Surg Am.

[16] Ripamanti CI, Santini D, Maranzano E (2012). Management of cancer pain: ESMO clinical practice guidelines. Ann Oncol.

[17] Leppert W, Buss T (2012). The role of corticosteroids in the treatment of pain in cancer patients. Curr Pain Headache Rep.

[18] Vyvey M (2010). Steroids as pain relief adjuvants. Can Fam Physician.

[19] Kaloostian PE, Yurter A, Etame AB (2014). Palliative strategies for the management of primary and metastatic spinal tumors. Cancer Control.

[20] Nomiya T, Teruyama K, Wada H (2010). Time course of pain relief in patients treated with radiotherapy for cancer pain. Clin J Pain.

[21] Maranzano E (2005). Short-course versus split-course radiotherapy in metastatic spinal cord compression: results of a Phase III, randomized, multicenter trial. J Clin Oncol.

[22] Silberman R, Roodman GD (2013). Myeloma bone disease: pathophysiology and management. J Bone Oncol.

[23] Boszczyk B (2010). Volume matters: a review of procedural details of two randomised controlled vertebroplasty trials of 2009.

[24] Fourney DR, Schomer DF, Nader R (2003). Percutaneous vertebroplasty and kyphoplasty for painful vertebral body fractures in cancer patients. J Neurosurg.

[25] De la Garza-Ramos R, Benvenutti-Regato M, Caro-Osorio E (2016). Vertebroplasty and kyphoplasty for cervical spine metastases: a systematic review and meta-analysis. Int J Spine Surg.

[26] Eleraky M, Papanastassiou I, Setzer M (2011). Balloon kyphoplasty in the treatment of metastatic tumors of the upper thoracic spine. J Neurosurg Spine.

[27] Berenson J, Pflugmacher R, Jarzem P (2011). Balloon kyphoplasty versus non-surgical fracture management for treatment of painful vertebral body compression fractures in patients with cancer: a multicentre, randomised controlled trial. Lancet Oncol.

[28] Markmiller M (2015). Percutaneous balloon kyphoplasty of malignant lesions of the spine: a prospective consecutive study in 115 patients. Eur Spine J.

[29] McDonald RJ, Trout AT, Gray LA (2008). Vertebroplasty in multiple myeloma: outcomes in a large patient series. AJRN Am J Neuroradiol.

[30] Pflugmacher R, Kandziora F, Schroeder J (2006). Percutaneous balloon kyphoplasty in the treatment of pathological vertebral body fracture and deformity in multiple myeloma: a one-year follow-up. Acta Radiol.

[31] Papanastassiou ID, Eleraky M, Murtagh R (2014). Comparison of unilateral versus bilateral kyphoplasty in multiple myeloma patients and the importance of preoperative planning. Asian Spine J.

[32] Kayanja MM, Schlenk R, Togawa D (2006). The biomechanics of 1, 2, and 3 levels of vertebral augmentation with polymethylmethacrylate in multilevel spinal segments. Spine.

[33] Hentschel SJ, Burton AW, Fourney DR (2005). Percutaneous vertebroplasty and kyphoplasty performed at a cancer center: refuting proposed contraindications. J Neurosurg Spine.

[34] Chi HK, Chung CK, Sohn S (2013). Less invasive palliative surgery for spinal metastases. J Surg Oncol.

[35] Zairi F, Arikat A, Allaoui M (2012). Minimally invasive decompression and stabilization for the management of thoracolumbar spine metastases. J Neurosurg Spine.

[36] Moussazadeh N, Rubin DG, McLaughlin L (2015). Short-segment percutaneous pedicle screw fixation with cement augmentation for tumor-induced spinal instability. Spine J.

[37] Gu Y, Dong J, Jiang X (2016). Minimally invasive pedicle screws fixation and percutaneous vertebroplasty for the surgical treatment of thoracic metastatic tumors with neurologic compression. Spine.

[38] Rosa MA, Maccauro G, Sgambato A (2003). Acrylic cement added with antiblastics in the treatment of bone metastases. J Bone Joint Surg Br.

[39] Maccauro G, Cittadini A, Casarci M (2007). Methotrexate-added acrylic cement: biological and physical properties. J Mater Sci Mater Med.

[40] Wallace AN, Greenwood TJ, Jennings JW (2015). Radiofrequency ablation and vertebral augmentation for palliation of painful spinal metastases. J Neurooncol.

[41] Gevargez A, Groenemeyer DHW (2008). Image-guided radiofrequency ablation of spinal tumors. Eur J Radiol Open.

[42] Pezeshki D, Davidson S, Murphy K (2016). Comparison of the effect of two different bone-targeted radiofrequency ablation (RFA) systems alone and in combination with percutaneous vertebroplasty (PVP) on the biomechanical stability of the metastatic spine. Eur Spine J.

[43] Zheng L, Chen Z, Sun M (2014). A preliminary study of the safety and efficacy of radiofrequency ablation with percutaneous kyphoplasty for thoracolumbar vertebral metastatic tumor treatment. Med Sci Monit.

[44] Bilsky M, Smith M (2006). Surgical approach to epidural spinal cord compression. Hematol Oncol Clin North Am.

[45] Laufer I, Iorgulescu JB, Chapman T (2013). Local disease control for spinal metastases following “separation surgery” and adjuvant hypofractionated or high-dose single fraction stereotactic radiosurgery: outcome analysis in 186 patients. J Neurosurg Spine.

[46] Tatsui CE, Stafford J, Li J (2015). Utilization of laser interstitial thermotherapy guided by real-time thermal MRI as an alternative to separation surgery in the management of spinal metastasis. J Neurosurg Spine.

[47] Tatsui CE, Lee SH, Amini B (2016). Spinal laser interstitial thermal therapy: a novel alternative to surgery for metastatic epidural spinal cord compression. Neurosurg.

[48] Tatsui CE, Belsuzarri TA, Oro M (2016). Percutaneous surgery for treatment of epidural spinal cord compression and spinal instability: technical note. Neurosurg Focus.

[49] Tatsui CE, Nascimento CN, Suki D (2017). Image guidance based on MRI for spinal interstitial laser thermotherapy: technical aspects and accuracy. J Neurosurg Spine.

